# Conservative Management of Uterine Fibroid-Related Heavy Menstrual Bleeding and Infertility: Time for a Deeper Mechanistic Understanding and an Individualized Approach

**DOI:** 10.3390/jcm10194389

**Published:** 2021-09-26

**Authors:** Marie-Madeleine Dolmans, Luciana Cacciottola, Jacques Donnez

**Affiliations:** 1 Gynecology Department, Cliniques Universitaires St-Luc, Avenue Hippocrate 10, 1200 Brussels, Belgium; marie-madeleine.dolmans@uclouvain.be; 2 Gynecology Research Unit, Institut de Recherche Expérimentale et Clinique, Université Catholique de Louvain, Avenue Mounier 52, 1200 Brussels, Belgium; luciana.cacciottola@uclouvain.be; 3 Université Catholique de Louvain, 1200 Brussels, Belgium; 4 Société de Recherche pour l’Infertilité (SRI), 143 Avenue Grandchamp, 1150 Brussels, Belgium

**Keywords:** fibroids, myomas, intramural fibroids, GnRH antagonist, infertility, medical therapy

## Abstract

(1) Background: Uterine fibroids are the most common form of benign uterine tumors, causing heavy menstrual bleeding (HMB), pelvic pain, infertility and pressure symptoms. Almost a third of women with uterine fibroids seek treatment. The objective of this review is to understand the mechanisms linking fibroids to these symptoms and evaluate different options for their management, particularly the place of gonadotropin-releasing hormone (GnRH) antagonist. (2) Methods: We gathered the most recent and relevant papers on the main fibroid-related symptoms and medical and surgical therapy for their treatment. Those reporting use of oral GnRH antagonists were investigated in detail. (3) Results: The mechanisms explaining myoma-related HMB and infertility were reviewed, as they are essential to a deeper mechanistic understanding and oriented approach. The choice of treatment depends on the number, size, and location of fibroids, and is guided by the patient’s age and desire to preserve her fertility. Economic impacts of myomas in terms of direct costs, lost workdays, and complications were found to be significant. Medical, surgical, and non-surgical strategies were analyzed in this context. Novel medical approaches with GnRH antagonist were explored and found to represent an effective new option. (4) Conclusion: The need for alternatives to surgical intervention is very real, especially for women seeking to preserve their fertility. New options now exist, with GnRH antagonists proven to treat fibroid symptoms effectively, opening the door to novel strategies for the management of myomas.

## 1. Introduction

**Figure 1:**
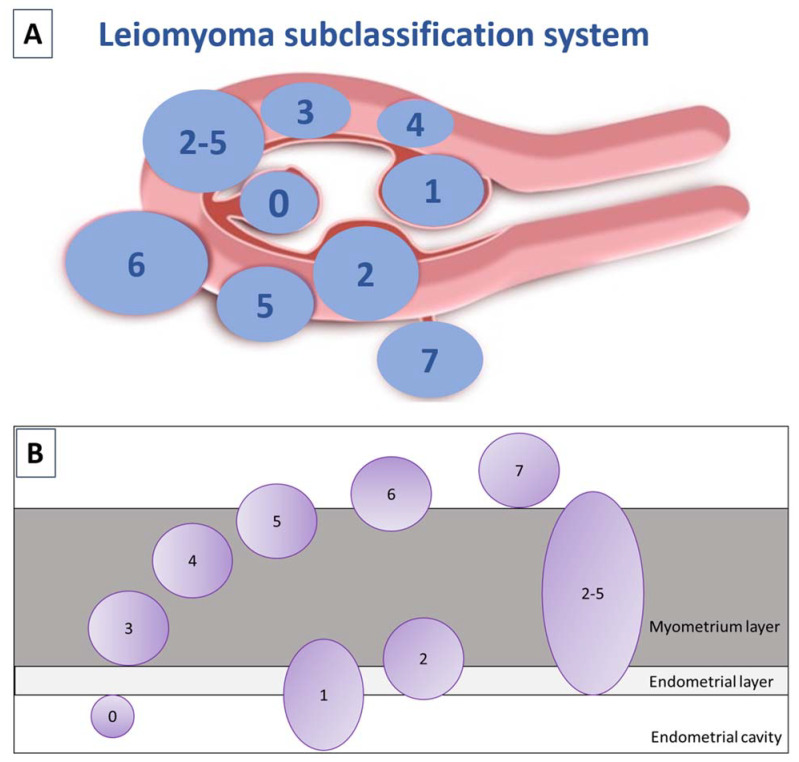
(**A**) FIGO subclassification of leiomyomas according to Munro et al., 2011. Fibroid types range from 0 to 8: 0 = pedunculated (intracavitary); 1 = submucosal (<50% intramural); 2 = submucosal (≥50% intramural); 3 = contact with endometrium (100% intramural); 4 = intramural; 5 = subserosal (≥50% intramural); 6 = subserosal (<50% intramural); 7 = subserosal (pedunculated); and 8 = other (cervical, parasitic). Where two numbers are given (2–5), the first number pertains to the relationship with the endometrium, and the second with the serosa, so 2–5 is both submucosal and subserosal, with less than half of its diameter in the endometrial and peritoneal cavities respectively. Diagram showing the classification system adapted from Munro et al. [9]. (**B**) Relationship between fibroids and the myometrium adapted by Donnez and Dolmans, 2020 [10].

Uterine fibroids are the most commonly encountered benign uterine tumors [1,2,3,4,5]. They occur in 50–60% of women, rising to 70% by the age of 50. Race and age have emerged as the most significant risk factors for their development [6]. African-American females, as well as those of African descent residing in Europe, are at higher risk of suffering from uterine fibroids at a younger age [6,7]. Early onset of menstruation, later first pregnancy, low parity, obesity, hypertension, caffeine and alcohol consumption, and some specific gene alterations are also linked to myoma growth [3,4,8]. Over the last 10 years, the International Federation of Gynecology and Obstetrics (FIGO) classification, has distinguished 8 types of fibroids, as well as a hybrid class that takes into account the degree of intramural extension and uterine cavity distortion (Figure 1) [9]. Vaginal ultrasound is recommended for identifying fibroids, but making a differential diagnosis of uterine masses is of crucial importance [10,11]. Differentiating adenomyosis from myomas can indeed be challenging. In case of ambiguous ultrasound findings, magnetic resonance imaging (MRI) may be used to shed more light.

In 30–40% of cases, uterine fibroids display a variety of symptoms, depending on their location and size. They can cause heavy menstrual bleeding (HMB) with subsequent anemia, which could be life-threatening. Large myomas can also result in pressure symptoms (bulk symptoms) and bladder dysfunction, including increased daytime urinary frequency and urinary incontinence [1,2,3,4,5]. Dysmenorrhea and pelvic pain are often encountered, impacting quality of life and undermining daily activities [12,13]. Abdominal distention and pelvic pressure on the ureters (causing hydronephrosis) may also interfere with quality of life [4,5,6,7,8,9,10,11,12,13]. Furthermore, uterine fibroids can cause infertility, depending on their location in the myometrium [12]. The two most troublesome complaints necessitating treatment during reproductive age are (i) HMB associated or not with pain [14,15], and (ii) infertility [12].

## 2. Heavy Menstrual Bleeding and Pain

Abnormal uterine bleeding (AUB) is a clinical entity. Classification according to the acronym PALM-COEIN (polyp, adenomyosis, leiomyoma, malignancy and hyperplasia, coagulopathy, ovulatory dysfunction, endometrial, iatrogenic, and not yet classified) allows a structured approach to establishing the cause of AUB [9]. HMB, a subgroup of AUB, is more closely related to the presence of myomas [2,4,9,13,14,15]. HMB is considered by Critchley et al. [16] as monthly blood loss of more than 80 mL. However, as pointed out later by Whitaker and Critchley [14], the Royal College of Obstetricians and Gynecologists (RCOG) and the American College of Gynecologists (ACOG) favor a patient-oriented definition, namely ‘excessive menstrual blood loss which interferes with a woman’s physical, social, emotional and/or material quality of life’, to indicate treatment options. Pain is common in women with uterine fibroids and frequently associated with HMB and passing of blood clots. The distress that women suffer from bleeding and pain is underestimated [17].

A century ago, women menstruated approximately 40 times over the course of their lifetime, owing to pregnancy and lactation amenorrhea. Now, in developed countries, women can expect up to 400 menses during their life [13]. They delay having children for a variety of reasons, such as personal choice or prioritization of career [12,13]. Consequently, AUB has become much more common. On the other hand, these women wish to preserve their uterus and fertility, so surgical options like hysterectomy are not appropriate and medical alternatives must be considered [4]. Cardozo et al. [18] estimated that annual direct and indirect economic costs linked to AUB were in the order of $1 and $12 billion respectively.

The relationship between HMB and fibroids remains poorly understood, particularly the understanding of endometrial function in women with structural myometrial features like leiomyomas. A number of theories have been proposed in the literature, as reported by Whitaker and Critchley [14] and Critchley et al. [16] in an excellent review written after a 2-day meeting seeking to ‘identify gaps and opportunities in menstruation science’.

**Figure 2:**
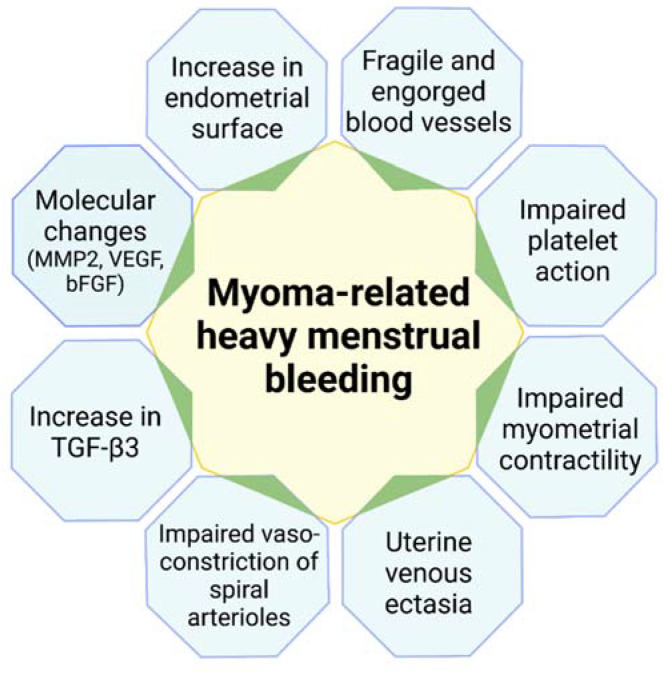
Mechanisms linking uterine fibroids and heavy menstrual bleeding. MMP-2: matrix metalloproteinase; VEGF: vascular endothelial growth factor; bFGF: basic fibroblast growth factor; TGF-β3: transforming growth factor-β3.

The mechanisms linking uterine fibroids and HMB are explained here in greater depth (Figure 2).

### 2.1. Increased Endometrial Surface Area

In 2012, when reviewing the classification of menstrual bleeding, Munro suggested that an increase in endometrial surface area and the uterine cavity could contribute to HMB [19].

### 2.2. Presence of Dilated Blood Vessels on the Myoma Surface

**Figure 3:**
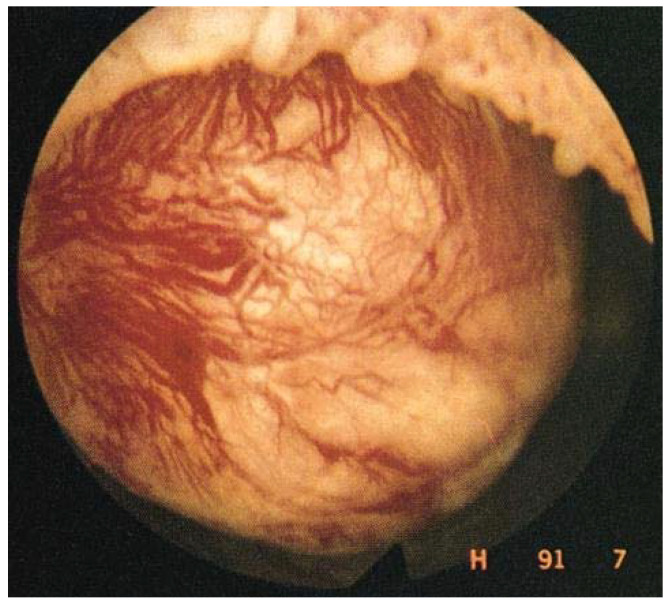
Enhanced vascularization with the presence of dilated blood vessels on the myoma surface, visible by hysteroscopy.

Enhanced vascularization can be seen by hysteroscopy performed at the time of menstruation [20]. Since the early 1990s, hysteroscopy has become a routine diagnostic tool, often combined with transvaginal ultrasound. During menstruation, active bleeding can be observed by pressure-controlled hysteroscopy [21] (Figure 3).

### 2.3. Uterine Venous Ectasia by Compression from the Myoma

In 1981, Buttram and Reiter suggested that uterine fibroids in various sites within the uterus cause venule ectasia by compressing veins [22]. Some years later, Stewart and Nowak suggested that these vascular anomalies were more likely the consequence of local action of vasoactive growth factors [23].

### 2.4. Platelet Action Overcome by Vascular Flow in Engorged Vessels

In ectatic venules, hemostatic actions of platelet and fibrin plugs may be overwhelmed by the enlarged diameter of vessels [23]. Many molecular changes occur that could impact angiogenesis and coagulation by alteration of vasoactive substrates and growth factors, as suggested by Stewart and Nowak in 1996 [23].

### 2.5. Increased Uterine Contractility and Peristalsis

Intramural myomas cause abnormal internal peristalsis, which could interfere not only with blastocyst implantation, but also with menstrual bleeding [24]. Dysfunctional uterine contraction may result from increased production of prostaglandin F2 (PGF2) [25]. Indeed, Miura et al. were able to demonstrate that PGF2 levels were higher in homogenates of myomas and surrounding myometrium compared to normal myometrium [25].

### 2.6. Alterations to Vasoconstriction of Spiral Arterioles

Infiltration by macrophages and increased concentrations of monocyte chemotactic protein 1 (MCP-1), an inflammation-related factor observed in the endometrium of women with submucosal and intramural myomas [25], may interfere with uterine contraction mainly governed by the functional zone, as well as spiral arteriole function.

### 2.7. Leiomyomas Secreting Transforming Growth Factor-β3 (TGF-β3), Inducing Bone Morphogenic Protein 2 (BMP-2) Resistance and Impairing Endometrial Receptivity

Sinclair et al. [26] and Taylor [27] found TGF-β3 levels to be elevated in myoma-conditioned media, causing repression of the BMP-2 receptor and ultimately lack of response to BMP-2. Myomas situated closest to the uterine cavity let more TGF-β3 reach endometrial cells and consequently impair endometrial receptivity [27].

### 2.8. Excess TGF-β Production Associated with Reduced Levels of Plasmin Activator Inhibitor 1 (PAI-1) and Antithrombin III

Leiomyoma-associated endometrium expresses less PAI-1, a fibrinolytic modulator, and thrombomodulin in vivo. PAI-1 expression is increased 4-fold in the presence of leiomyomas. Sinclair et al. [26] suggested that elevated levels of PAI-1 expression may contribute to impaired hemostatic processes in women with fibroids, resulting in menorrhagia.

### 2.9. Increase in Fibroid Matrix Metalloproteinase (MMP) Levels

Protein expression levels of MMP-2 and MMP-9 were evaluated in leiomyoma tissue. MMP-2 activity was significantly higher in leiomyomas than normal myometrium [28], but its impact on endometrial bleeding remains unclear [14].

### 2.10. Changes in Expression of Potential Angiogenic Growth Factors

As reported by Whitaker and Critchley [14], expression of potential angiogenic factors, like vascular endothelial growth factor (VEGF), basic fibroblast growth factor (bFGF) and platelet-derived growth factor (PDGF), is altered in women with fibroids, but their specific role still needs to be determined.

## 3. Infertility

**Figure 4:**
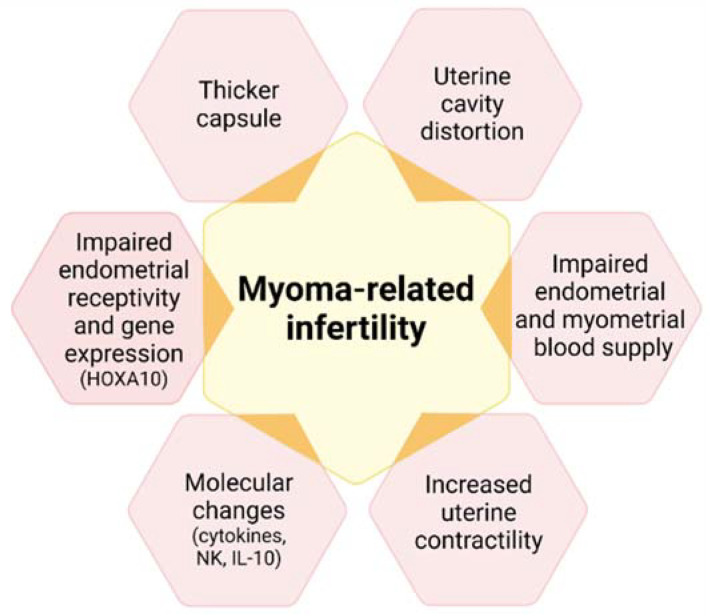
Mechanisms associating uterine fibroids with infertility. HOXA10: homebox A10; NK: natural killer; Il-10: interleukin 10.

Infertility and recurrent miscarriage may also be symptoms of fibroids, especially submucous and intramural myomas, which distort the uterine cavity [3,4,11,29]. The mechanisms linking uterine fibroids and infertility are indeed diverse (Figure 4), including uterine cavity distortion (fibroid types 0, 1, 2, 2–5), impaired endometrial/myometrial blood supply, greater uterine contractility, hormone, paracrine and molecular alterations, defective endometrial receptivity and gene expression (drop in homeobox A [HOXA] expression), and a thicker capsule [15].

### 3.1. Uterine Cavity Distortion

The first mechanism is clear to see and has been widely documented [30]. In the case of submucous fibroids, implantation, clinical pregnancy and live birth rates were found to be significantly lower than in control patients (without submucous myomas), while the spontaneous abortion rate was significantly higher.

### 3.2. Impaired Endometrial and Myometrial Blood Supply

The presence of fibroids close to the uterine cavity (type 3) interferes with endometrial blood flow. In a prospective study, Niewenhuis et al. [31] showed that the increase in myoma volume was greater in highly vascularized myomas, strongly supporting the notion that blood supply modifications may affect blastocyst implantation, as suggested by Schild et al. [32] and Kim et al. [33].

### 3.3. Increased Uterine Contractility

One study using MRI demonstrated that intramural myomas induced abnormal uterine peristalsis, resulting in lower implantation and pregnancy rates [34]. The same team reported that myomectomy decreases abnormal uterine peristalsis and increases pregnancy rates [35]. According to Fanchin et al. [36], uterine contractility diminishes in response to progesterone to favor embryo implantation. If the presence of intramural myomas alters uterine peristalsis, it may also affect the surrounding myometrium and lead to impaired uterine contractility.

### 3.4. Hormonal, Paracrine and Molecular Changes

As stressed by Ikhena and Bulun [37] and Vannuccini et al. [29], fibroids modify expression of genes important to implantation, such as glycodelin and BMP receptor type 2 (BMPR2), and significantly impact function and gene expression in endometrium.

### 3.5. Impaired Endometrial Receptivity and Gene Expression

According to Rackow and Taylor [38], endometrial expression of HOXA-10 (an important gene governing endometrial receptivity) is lower in the presence of submucous myomas. In 2010, this group suggested that endometrial receptivity was altered through a specific molecular mechanism of action, mediated by a molecule originating from the myoma [38]. It is possible that the same signaling pathway proceeds from intramural myomas to the endometrium, but has a less pronounced effect on endometrial receptivity [26,27]. The same groups subsequently showed that TGF-β3 is elevated in leiomyoma-conditioned media, leading to repression of BMP receptor types 1B and 2 and eventually a lack of response to BMP-2. They found that TGF-β operates as a diffusible signaling molecule to alter BMP-2, curtailing HOXA-10 expression throughout the endometrium and thereby interfering with implantation [27]. Focusing on both size and distance, Taylor suggested that larger fibroids generate more TGF-β3, while those closest to the uterine cavity allow more TGF-β to access endometrial cells [27]. The amount of TGF-β3 reaching the uterine cavity therefore varies by the square of the distance between the endometrium and the myoma [27].

### 3.6. Thicker Capsule

It is unclear whether an increased pseudocapsule thickness also boosts neuroendocrine fiber numbers, but their presence may affect muscle contractility and uterine peristalsis [39].

## 4. Non-Cavity-Distorting Uterine Fibroids and Infertility: Conclusive Remarks from Recent Literature Reports and Meta-Analyses

An extensive review was recently published by Donnez and Dolmans [12], who found that all published studies and meta-analyses agree that intramural myomas of more than 3 cm in size impair fertility, even if they do not distort the uterine cavity. In the present paper, the literature was limited to the two latest meta-analyses to avoid plagiarism with our previous paper [12]. In a meta-analysis of 28 studies involving 9189 patients, Wang et al. [40] reported that intramural myomas significantly reduced blastocyst implantation and live birth rates. Among 15 studies reviewed by Rikhraj et al. [41], 8 were prospective and recorded live birth rates. These systematic reviews found that women with non-cavity-distorting intramural fibroids undergoing in vitro fertilization (IVF) had a 44% lower chance of a clinical pregnancy than women without fibroids. In their review on myoma-related infertility, Donnez and Dolmans [12] found that non-cavity-distorting intramural fibroids do indeed have a deleterious impact on IVF outcomes. Two factors were significant, namely the size of myomas and the proximity of the uterine cavity [12]. As reported by Yan et al. [42], a type 3 myoma measuring 2 cm or more situated close to the endometrial lining will have a detrimental effect. As stressed very recently by Freytag et al. [11], although intramural myomas are reported to be associated with poorer pregnancy outcomes than in women without myomas, studies addressing the question of improved conception capacity after myomectomy are few and far between. A recent Cochrane review [43] failed to provide any definitive information or conclusions on this specific question for this very reason.

## 5. Apposite Medical Treatment

An appropriate strategy involving a deeper mechanistic understanding of menstruation and AUB was strongly advocated by numerous experts in a recent paper [13]. The same strategy should be applied in the context of myoma-related infertility [16]. Some investigators have recommended surgically removing intramural fibroids [27], but in their review, Donnez and Dolmans challenged this proposition [12]. To put it simply, if the negative effect is linked to myoma size and proximity of the uterine cavity, why not attempt a medical approach to shrink the size of the fibroid and push it further into the myometrium, something we call the ‘migration effect’ [12]? In a very recent ‘Fertile Battle’, Dolmans et al. [44] discussed the pros and cons of removal of symptomatic intramural myomas prior to IVF and concluded that reaching a consensus would not be easy [45].

As shown in Table 1, several medical treatments have been proposed for the management of uterine fibroids.

### 5.1. Oral Contraceptives/Progestogens

While oral contraceptives and progestogens may curtail AUB in case of moderate disease, they do not reduce myoma size, and therefore have limited benefits for women with fibroid-related infertility. Moreover, it is clear that progesterone and progestogens promote myoma growth via several signaling pathways [1,2,3,4,5,46]. In a recent review, the absence of evidence on the effectiveness of treating premenopausal women with uterine fibroids with progestogens was clearly demonstrated [5].

### 5.2. Tranexamic Acid

Tranexamic acid significantly reduces blood loss compared to a placebo, but has no impact on fibroid volume [47].

### 5.3. Levonorgestrel-Releasing Intrauterine System

The levonorgestrel-releasing intrauterine system (Mirena LNG-IUS) significantly abates menstrual bleeding, but fibroid volume reduction remains limited. Moreover, high expulsion rates are reported in case of submucous fibroids [48].

### 5.4. Selective Progesterone Receptor Modulators

The advantages of selective progesterone receptor modulators (SPRMs) have been clearly demonstrated in various studies [49,50,51]. They include a reduction in fibroid volume over 50% after two 3-month courses, marked and rapid control of bleeding, as well as restoration of hemoglobin levels.

Regrettably, the Pharmacovigilance Risk Assessment Committee of the European Medicines Agency (EMA) has laid down very strict indications for ulipristal acetate (UPA), an SPRM. The European Commission concluded (January 2021) that 5 mg UPA can be used for intermittent treatment of moderate-to-severe symptoms of uterine fibroids in adult women who have not reached menopause, if fibroid embolization and/or surgical treatment are not suitable options or have failed. This follows in the wake of a 2018 EMA review of five liver injury cases that required transplantation [52,53].

### 5.5. Gonadotropin-Releasing Hormone Agonists

Preoperative administration of gonadotropin-releasing hormone (GnRH) agonists (leuprolide, goserelin, triptorelin) boosts hemoglobin levels and significantly decreases fibroid volume, but long-term treatment is contraindicated because of menopausal symptoms, like bone mineral density (BMD) loss and hot flushes [54,55,56]. For over three decades now, GnRH agonists have been widely used to reduce the size of type 1 and 2 myomas prior to hysteroscopic resection [54].

### 5.6. GnRH Antagonists

**Figure 5:**
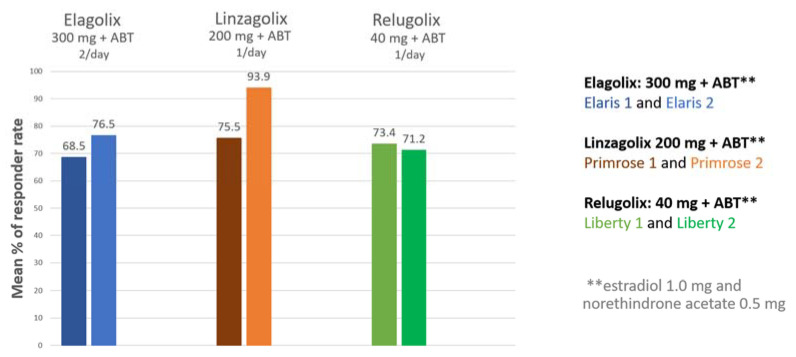
Percentage of women meeting the primary endpoint (those with menstrual blood loss of less than 80 mL and at least 50% down from baseline) in patients treated by a GnRH antagonist combined with add-back therapy (ABT) in the first and second clinical trial respectively (300 mg elagolix twice daily + ABT (Elaris 1 and 2); 200 mg linzagolix once daily + ABT (Primrose 1 and 2); 40 mg relugolix once daily + ABT (Liberty 1 and 2)).

Data from phase 3 clinical trials investigating oral GnRH antagonists (elagolix, relugolix, linzagolix) are now available [5,56,57,58,59,60]. Subjects received GnRH antagonist with add-back therapy [ABT] (1 mg estradiol + 0.5 mg norethisterone acetate). The results demonstrated excellent control of fibroid-related HMB and showed the reduction in bleeding to be maintained when ABT was associated, curbing BMD loss. Indeed, more than 70% of participants met the primary endpoint (menstrual blood loss <80 mL and >50% reduction from baseline) (Figure 5) and over 50% were amenorrheic [5].

**Figure 6:**
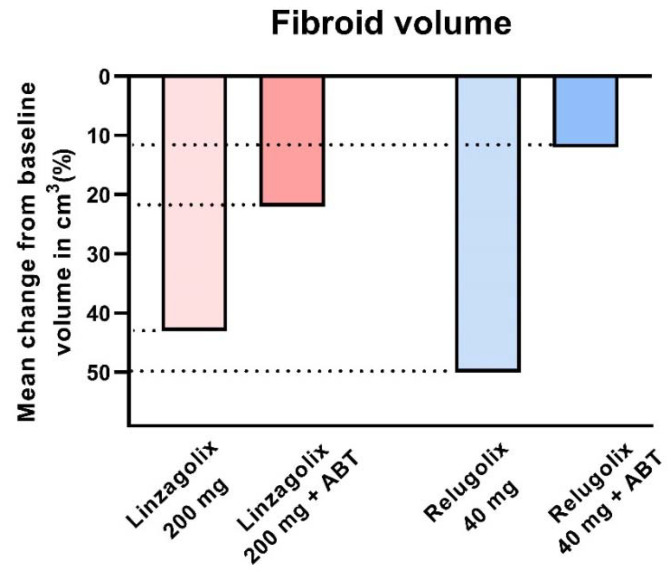
Fibroid volume reduction: 200 mg linzagolix and 40 mg relugolix significantly reduce myoma volume (*p* < 0.001). The decrease was not significant when ABT was added.

**Figure 7:**
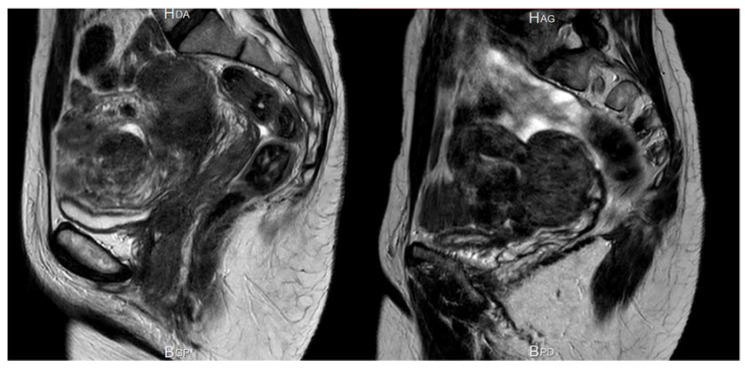
Significant reduction in myoma and uterine volume after 12 weeks of 200 mg/day linzagolix. At baseline, volume was estimated to be 396 cm^3^. After 12 weeks of treatment, it dropped to 169 cm^3^.

The decline in fibroid volume was, however, found to be more limited in subjects with ABT than without ABT. Indeed, Osuga et al. clearly demonstrated that 40 mg/day relugolix decreases fibroid volume by more than 50% after 24 weeks of treatment [57]. Steward et al. also concluded that 200 mg linzagolix reduces fibroid volume more efficiently than linzagolix + ABT (Figure 6 and Figure 7) [60]. Further studies are nevertheless needed to identify the best protocol and dose to use if reducing myoma volume is the intended goal, as in the case of myoma-related infertility.

## 6. Current Surgical and Non-Surgical Management Strategies

Conservative surgical and non-surgical approaches include myomectomy by hysteroscopy, myomectomy by laparotomy or laparoscopy, uterine artery embolization (UAE), and other interventions performed under radiological or ultrasound guidance [4].

### 6.1. Hysteroscopic Myomectomy

Advances in techniques and instruments have promoted hysteroscopic myomectomy to the rank of a standard minimally invasive procedure for submucous myomas [61,62,63]. Small fibroids (<2 cm) are routinely removed in an outpatient setting [63]. The most commonly used approach is the slicing technique. Repeated and progressive passage of a cutting loop allows the myoma to be cut into small chips until the fasciculated fibers of the myometrium are visualized [43,63,64].

If the myoma is large (>3 cm in diameter), there is an increased risk of intraoperative complications like perforation and/or damage to surrounding myometrium and fluid intravasation. In this case, use of preoperative GnRH antagonist therapy may facilitate surgery by significantly reducing the myoma size [49,50]. After resection of the protruded portion of the myoma, the residual intramural component rapidly migrates to the uterine cavity and can be resected during the same procedure or in a second step. Hysteroscopic myomectomy is effective for control of bleeding and enhancing fertility prospects [44], but failures are usually related to incomplete treatment of large intramural (partially submucous) myomas, growth of fibroids in other sites, or association of fibroids with adenomyosis [63].

In the previously mentioned ‘Fertile Battle’ [44], Zhang and Isaacson reported that infertile women showed improved clinical pregnancy rates after resection of submucosal fibroids, but recommendations for myomectomy are less clear for asymptomatic infertile patients with intramural fibroids that do not distort the endometrial lining (type 3–4) [44]. These authors maintain that removal of intramural myomas should be considered in women with infertility seeking assisted reproductive technology (ART). The size and location of intramural fibroids likely contributes to the success of ART, so emphasis should be placed on counseling women about myomectomy for type 3 fibroids measuring 2 cm or more as first-line therapy [44].

### 6.2. Laparoscopic Myomectomy

The advantages of laparoscopic myomectomy over laparotomy are well known, namely less severe postoperative morbidity, faster recovery, and no significant difference between reproductive outcomes after laparoscopic or abdominal myomectomy [43,65,66,67]. Contraindications to laparoscopic myomectomy typically include the presence of an intramural myoma measuring >10–12 cm in size or multiple myomas (≥4) in different sites of the uterus, requiring numerous incisions [67]. During laparoscopic myomectomy, leiomyomas are removed with a morcellator inside (or not) a bag or through the cul-de-sac of Douglas, or by minilaparotomy to avoid the threat of dispersing tissue fragments. The risk of uterine fragment dispersion, with subsequent appearance of pelvic adenomyotic masses and parasitic leiomyomas, was first described in 2006 [68] and remains a concern that may be avoided by extensive peritoneal lavage and careful removal of all the fragments. On the other hand, the Food and Drug Administration (FDA) has issued warnings about use of electromechanical power morcellation [69,70,71]. It should be stressed, however, that the prevalence of sarcoma in leiomyomas is <0.3% and the debate around electric morcellation has probably been somewhat inflated, not only because of fear of medico-legal problems, but also for emotional reasons. The technique of power morcellation in a bag does minimize the risk of inadvertent tissue spread, but there is no evidence that this technique will not increase postoperative complications [72]. In some rare cases, histology may reveal the presence of a uterine smooth muscle tumor of uncertain malignant potential (STUMP), also presenting a challenge in terms of fertility preservation. A recent study of 57 patients [73] with STUMP suggested that a fertility-sparing approach is feasible, but patients should be informed about the risk of recurrence (14% in the series of Şahin et al.) and poor prognosis of recurrent STUMP [73]. The authors strongly advocate performing complementary surgery after successful pregnancy, if a fertility-sparing technique was used.

In the same ‘Fertile Battle’ [44], Gordts stressed the continued absence of consensus. Indeed, he noted that over 150 years after the first reported successful abdominal myomectomy in 1845 by brothers Washington and John Atlee, experts are still debating the advantages of myomectomy and its impact on reproductive performance. The localization of the myoma in relation to the junctional zone plays a crucial role in implantation and deep placentation. Intramural myomas have a negative impact on reproductive and obstetric outcomes, showing an improvement in terms of fertility after myomectomy [44]. Concerning the surgical approach, Gordts remains cautious, stating that a ‘decision for a laparoscopic approach must be balanced between the uterine pathology and the experience of the surgeon’, as there is much more to gain for patients and surgeons from a well performed myomectomy by laparotomy than a difficult laparoscopy with inappropriate suturing [44].

### 6.3. Laparoscopic Cryomyolysis and Thermocoagulation

Laparoscopic cryomyolysis and thermocoagulation both have the same goal, which is to reduce or suppress the primary blood supply and induce myoma shrinkage by causing sclerohyaline degeneration (at very low or very high temperatures). For cryomyolysis, a cryoprobe is inserted into the myoma and cooled to a temperature of <90°C. For laparoscopic thermocoagulation, either a monopolar or bipolar probe is placed inside the myoma before delivering an electrical current. Results in terms of success rates are contentious [74].

### 6.4. Uterine Artery Embolization

This technique, first used by Ravina in 1995 [75], triggers ischemic necrosis in fibroids, while the myometrium revascularizes. Most fibroids are targeted simultaneously. Although UAE is highly effective for treating symptoms (reduction in bleeding and myoma size), the risk of reoperation is a legitimate concern, reaching rates of 15–20% after successful embolization and up to 50% in case of incomplete infarction [74,75,76]. A systematic review of pregnancy outcomes after fertility-sparing treatment of uterine fibroids reported high rates of successful pregnancies after myomectomy (75.6%), while post-UAE conceptions yielded the lowest live birth rates (60.6%) and highest miscarriage rates (27.4%) [77]. According to a recent paper [78], fibroid-related quality of life two years post-treatment was better in women who underwent myomectomy than those undergoing UAE.

### 6.5. High-Frequency Magnetic Resonance-Guided Focused Ultrasound Surgery

High-frequency magnetic resonance-guided focused ultrasound surgery (MRgFUS) is thermal ablation using MRI to visualize myomas and define the target. Ultrasonic energy is directed at a point inside the fibroid and coagulation tissue necrosis is induced. In theory, damage to surrounding tissue is minimal. However, a systematic review by Verpalen et al. [79] reported that the quality of evidence on improved symptoms was poor-to-moderate, and the rate of reintervention reached more than 20% in some series.

## 7. Why Do We Need New Algorithms?

Fibroids are highly prevalent and constitute a heavy health burden [11,12]. Indeed, about 30% of women with leiomyomas will request treatment due to morbidities like HMB, abdominal pain, pressure symptoms and/or infertility [4]. Current therapies are mainly surgical and expensive. Among 600,000 hysterectomies performed each year in the USA, 200,000 are for fibroids. In a study by Flynn et al. [80], health care costs for management of leiomyomas were estimated to be over $2 billion a year, and even more when indirect costs are taken into account. There is no doubt that fibroids have a significant economic impact and markedly affect quality of life. According to Chadankar and Critchley [81], available evidence suggests that levels of satisfaction with current treatment options are poor, often resulting in women opting for major surgery like hysterectomy. It is therefore necessary to individualize the medico-surgical strategy according to the symptoms and wishes of the patient. It is time for a tailored approach based on the main symptoms (HMB, infertility) and what the patient really wants: a symptom-oriented approach.

**Figure 8:**
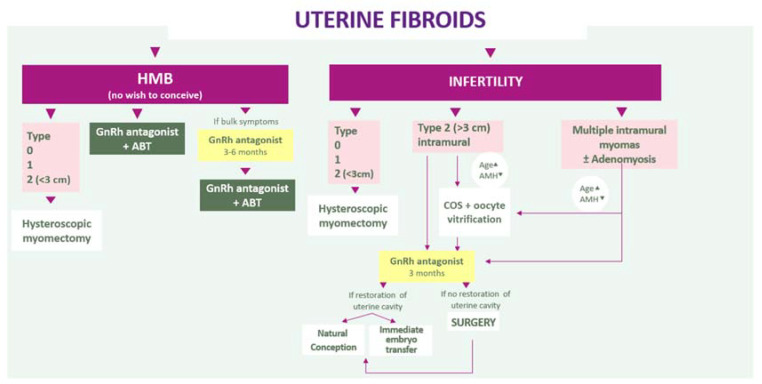
Algorithms that consider both myoma type and the most troublesome symptoms (HMB associated or not with pain or infertility).

According to the results of 3 randomized controlled trials, oral GnRH antagonists (elagolix, relugolix, linzagolix) allow control of uterine bleeding and associated pain, and improve quality of life. When administered without ABT, they significantly reduce fibroid size. For this reason, GnRH antagonists alone for a defined period of 3–6 months may be considered a first-choice treatment in case of HMB with bulk symptoms, followed by GnRH therapy with ABT, in order to maximize the myomas’ volume reduction. Moreover, GnRH antagonist without ABT for a short period of 3 months may help restore distorted uterine cavities responsible for infertility and decrease the need of uterine surgery. This means we can propose new algorithms that consider both myoma type (according to the FIGO classification) and the most troublesome symptoms (HMB associated or not with pain or infertility) (Figure 8).

These algorithms warrant investigation and confirmation by future clinical trials. Appropriate counseling is essential and health care providers need to tailor the ideal treatment to each and every woman. We cannot overlook the costs of new medical options, but neither can we ignore the costs linked to fibroids. It is vital that we promote research and evaluate new strategies in real-world populations.

## Figures and Tables

**Table 1:** Advantages and disadvantages of various medical therapies for intramural myomas.

Treatment Type (Medical Treatment)	Advantages	Disadvantages
Estroprogestestogens	May reduce AUB in case of moderate disease.	Absence of fibroid volume reduction.
Tranexamic acid/mefenamic acid	Reduces HMB in women without uterine fibroids; improves health-related quality of life.	Impact on fibroids unknown.
LNG-IUS	Treatment of choice for HMB in the absence of fibroids; provides contraception.	Cannot be used if the uterine cavity is distorted by fibroids; high expulsion rate with submucosal fibroids.
SPRMs	Curtail HMB and shrink fibroids; not associated with menopausal side effects or bone demineralization (restricted indications by the European Medicines Agency [EMA]).	Associated with endometrial alterations known as progesterone receptor modulator-associated endometrial changes; require liver enzyme monitoring (restricted indications).
Mifepristone	Able to reduce bleeding and pressure symptoms for up to 6 months.	Uncertain impact on fibroid volume.
GnRH agonists	may be given for 3–6 months before surgery to decrease uterine and fibroid size; serve to correct iron deficiency anemia.	Long-term treatment beyond 6 months can reduce bone density; vasomotor and other menopausal symptoms are common.
GnRH antagonists	Fast effect on HMB; reduce fibroid volume and correct anemia; dose-dependent efficacy and side effects; low doses cause limited loss of bone mineral density.	High doses erode bone mineral density, so require add-back therapy for long-term treatment; other menopausal symptoms commonly observed at high doses.

## Data Availability

Not applicable.
